# Post pubertal outcome after use of oral mucosa in urethral reconstruction for hypospadias in prepubertal boys: a systematic review

**DOI:** 10.1007/s00383-025-05982-3

**Published:** 2025-02-13

**Authors:** George Tsachouridis, Rien Nijman, Laetitia M. O. de Kort, Petra de Graaf

**Affiliations:** 1 Department of Urology, University Medical Center Utrecht, Utrecht, The Netherlands; 2 Department of Pediatric Urology, University Medical Center Utrecht, Utrecht, The Netherlands; 3 Department of Urology, University Medical Center Groningen, Groningen, The Netherlands; 4 Regenerative Medicine Center Utrecht, HP C04.236, P.O. Box 85500, 3508 GA Utrecht, The Netherlands

**Keywords:** Systematic review, Hypospadias, Oral mucosa graft, Puberty, Reconstructive surgery

## Abstract

Oral mucosa graft urethroplasty (OMGU) is a surgical technique used in complex hypospadias repair. However, long-term outcomes in prepubertal boys, particularly through and after puberty, are not well established. To systematically review the long-term outcomes of OMGU in prepubertal boys with hypospadias, focusing on changes observed after puberty. A systematic literature search was conducted in PubMed, Embase, and Cochrane Library. Studies reporting long-term outcomes of OMGU in prepubertal boys with hypospadias were included. The quality of evidence was assessed using the ROBINS-I tool. Three studies of moderate quality met the inclusion criteria. Outcomes were mixed, with some patients experiencing favorable results and others facing complications such as meatal stenosis, urethral strictures, and fistulas. The scarcity of high-quality, long-term studies highlights a significant knowledge gap in this area. This systematic review reveals insufficient evidence to establish the long-term efficacy and safety of OMGU in prepubertal boys with hypospadias, particularly concerning pubertal development and post-pubertal outcomes. Future research should prioritize prospective, longitudinal studies with larger sample sizes and standardized outcome measures to address these critical gaps in understanding.

**Supplementary Information:**

The online version contains supplementary material available at 10.1007/s00383-025-05982-3.

## Introduction

Urethral reconstruction in prepubertal boys presents a complex challenge in pediatric urology, particularly in cases of severe hypospadias and failed hypospadias surgery. One of the most significant problems faced by surgeons in resolving these cases is the lack of suitable tissue for urethroplasty. While many techniques have been developed in the last 40 years, none can create more tissue to correct this demanding situation.

Over the last 20 years, researchers have used more autologous tissue from various sources to overcome this problem. The preputial and/or hairless genital skin is the first choice of graft for urethral reconstruction. However, in certain complex urethral reconstructions, including hypospadias repair cases, suitable local tissue may not be available. Several extragenital tissue alternatives, with varying results, have been described.

The oral mucosa has gained popularity due to its thin and highly vascular lamina propria for bridging urethral defects and treating urethral strictures in adults and children. Oral mucosa has been successfully used to correct urethral strictures in adults [1].

Long-term follow-up (FU) is mandatory in hypospadias surgery and urethral reconstruction to evaluate the results of the technique and tissue used, especially when performed at (prepubertal) young age. During puberty, non-genital tissue used for urethroplasty has to keep up with the accelerated growth of penile tissue induced by the rise in testosterone levels.

Even in the hands of experienced pediatric urologists, the complication rate of hypospadias repair remains relatively high, with an average of 10–50% at long-term follow-up, depending on the severity of hypospadias and the surgical technique [2, 3]. The results show that more complications occur after a longer postoperative interval. Therefore, the outcome of hypospadias surgery can only be evaluated once the patient reaches and passes puberty [4, 5].

The purpose of this systematic review is to search the literature for articles that have a long-term follow-up of patients who underwent urethral reconstruction for hypospadias using oral mucosa before puberty and followed up until after puberty.

## Material and methods

### Literature search

A systematic review of the literature was performed in the PubMed and Embase databases and Cochrane Library databases, in June 2024 following the Preferred Reporting Items for Systematic Reviews and Meta-Analyses (PRISMA) guidelines [6]. The protocol was registered in Prospero under number CRD42023405977. The search terms included: urethral reconstruction, hypospadias, use of oral mucosa or use of buccal mucosa, pediatric patients, long term follow-up. The search was limited to the English language.

The PRISMA checklist was used to ensure comprehensive reporting of the review process.

A PRISMA flow diagram was created to illustrate the study selection process, including the number of records identified, screened, assessed for eligibility, and included in the final analysis. The complete PRISMA checklist as well as a full list of search terms are included in supplemental materials (S1).

### Study selection and risk of bias analysis

The results were imported into Rayyan QRCI software, and duplicates were removed. The screen based on the title and abstract was performed by two authors independently (GT, PdG); the full-text screen was performed by the same two authors (GT, PdG), also independently. Any differences in the screening results were solved by discussion.

**Fig. 1:**
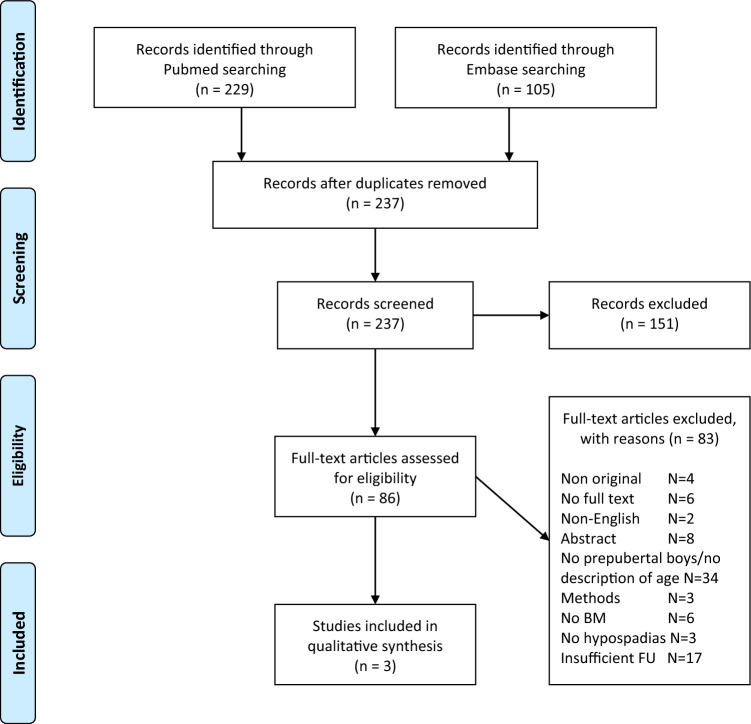
PRISMA flow diagram

Exclusion criteria were languages other than English, non-original papers (abstract, comment or review paper), age of the population unclear or post-pubertal (wrong population), short follow-up, and preclinical studies (wrong study design). The flow diagram of the process is depicted in Fig. 1.

To assess methodological quality of the included studies, we performed a risk of bias (RoB) analysis using the ROBIN-I for clinical studies [7].

The ROBINS-I tool evaluates seven domains of bias: confounding, selection of participants, classification of interventions, deviations from intended interventions, missing data, measurement of outcomes, and selection of the reported result. Each domain is rated as low, moderate, serious, or critical risk of bias. The overall risk of bias for each study is determined based on the highest risk of bias in any domain.

### Data extraction

After study selection, we retrieved relevant outcomes from all articles (number of patients, follow up and outcome). Regarding clinical studies, we defined the use of oral mucosa successful when no reoperation was needed. We did not combine outcomes in a meta-analysis because of the small number of papers included, as well as extensive methodological differences between studies.

## Results

After a systematic literature search, only three articles met the inclusion criteria (Table 1 and Fig. 1).

**Table 1:** Data extracted from the systematic analysis

Author	Type of study	Number of patients	Surgical approach	Years of follow up	Fistulas	Meatal Stenosis	Stricture	Recurrent Curvature	Micturition	Cosmesis
Nelson et al.	Retrospective	43	Tube graft (7), on-lay graft the rest	Mean 15.1 years	22	5	6	Not specified	Persistent urinary spray (11), difficulties in directing urinary stream (12)	Not specified
Figuera et al.	Retrospective	137 (10 included)	Staged buccal mucosa graft urethroplasty	10	No	1	No	No Recurrence in the long term Ventral penile curvature (3), graft contraction requiring regrafting (1)	Not specified	Varied final meatal position (glandular in 9, coronal in 1)
Goyal et al.	Retrospective	37	Not specified	Median 9.5 years	7	5	2	Not specified	Not specified	Not specified

Nelson et al. conducted a cross-sectional study involving 66 patients who underwent oral mucosa graft urethroplasty (OMGU) procedures between 1992 and 2003 [8]. Out of 51 patients that could be traced, 43 patients agreed to participate (84%). The study instrument, consisting of 20 items, was a combination and modification of validated questionnaires. It included the American Urological Association (AUA) International Prostate Symptom Score (IPSS) index for Benign Prostatic Hyperplasia (BPH) to assess urinary function, supplemented by two additional items from previous studies to evaluate urinary spraying and urinary stream. Oral symptoms were also analyzed but were not included in our study. The assessment focused on satisfaction with urinary function, penile appearance, and surgical results. Mean age at the time of the study was 15.1 ± 11.2 years. Age categories were 11 years or younger (51%), 11–17 years (23%),18 years and older (26%). All patients were operated on before puberty. Complaints regarding urinary spraying were reported by 11 patients (26%), while 12 patients (28%) experienced problems with aiming the stream. In terms of the AUA symptom index score IPSS, the majority of patients (77%) had mild symptoms, with 16% reporting moderate symptoms, and 5% reporting severe symptoms.

Patients who had undergone OMGU after a previous failed urethral repair tended to have a worse IPSS. Overall satisfaction with urinary function was mixed, with 60% of patients feeling mostly satisfied or better. Urethral complications were common post-OMGU, with 22 patients (51%) requiring one or more additional procedures. Among these patients, five had meatal stenosis, six had urethral strictures, five had urethrocutaneous fistulas, and six had a combination of these problems.

In the retrospective study conducted by Figueroa et al., the medical records of 137 patients who underwent oral mucosa urethroplasty between 2000 and 2010 were examined [9]. Further analysis was conducted specifically on boys who underwent the first stage of the procedure before the age of 12 and had their last follow-up recorded after puberty. The data reviewed included demographic information, initial meatal location, quality of the graft before tubularization, flow rate, and complications. Out of the initial 137 patients, only 10 met the inclusion criteria for this study due to lack of agreement, missing follow-up data, or failure to meet the inclusion criteria. The mean age of patients at the time of surgery was 8 years old (range 5–11 years), with a mean follow-up period of 40.6 months (range 9–66 months). Among these patients, 5 had undergone a redo procedure following failed hypospadias correction using the urethral plate (TIP). Complications observed included one fistula and two cases of glandular dehiscence. The final position of the meatus was glandular in 9 patients and coronal in one. No cases of ventral curvature were reported by neither the patients nor their parents. The average maximum flow rate, as reported by the authors, was considered adequate for the patients’ age, with a mean of 25.7 ml/sec.

In the retrospective study conducted by Goyal et al., out of the initial cohort of 37 patients who underwent OMGU between 1994 and 2002, 30 patients were available for inclusion in the study [10]. Among these, 28 were categorized as post pubertal at the time of the study, while the remaining 2 were classified as peripubertal. Early surgical complications were observed in 10 patients, with the most common complications being fistula formation in 7 cases, urethral stricture in 2 cases, and a single case of tortuous urethra. In terms of long-term complications, one patient developed obstructive balanitis xerotica obliterans in the grafted urethra and 5 meatal stenosis.

### Risk assessment ROBINS-I

Table 2 provides an overview of the risk of bias assessment for each included study using the ROBINS-I tool.

**Table 2:**
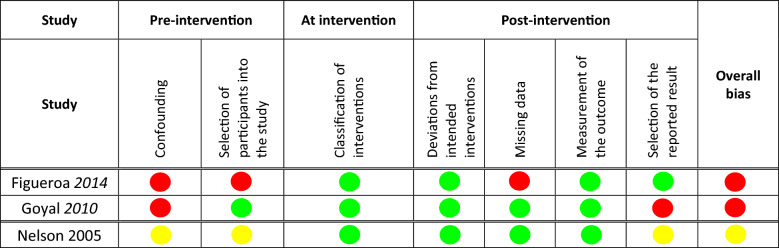
Outcomes RoB analysis using ROBINS-I (7)

Most bias occurs prior or after the intervention, whereas the intervention itself is without bias. The study of Figueroa et al. [9] lacked a control group, selection was unclear and the high percentage of participants excluded due to missing data raised critical risks of bias in these aspects. Despite these limitations, the study demonstrated low risk in the classification of interventions and deviations from intended interventions. Overall, this study’s risk of bias is assessed as serious. Similar to the previous study, the study of Goyal et al. [10] lacked a control group or comparison with other techniques introduces a serious risk of bias due to confounding. Furthermore, the use of non-validated questionnaires to assess outcomes and the method of retrospective assessment of files with different severity of hypospadias led to a serious risk of bias in outcome measurement. Despite the low risk of bias in the selection of participants and classification of interventions and that no deviations from intended interventions were documented nor missing data, the overall risk of bias in this study is serious. The last study, Nelson et al. [8], displayed an overall moderate risk of bias. There was moderate risk due to confounding and the lack of explicit information on participant selection and on the self-reported questionnaires for outcome measurement. On the other hand, the classification of interventions demonstrated a low risk of bias, with a clear description of the surgical techniques and deviations from intended interventions and missing data.

## Discussion

This systematic review aimed to evaluate the outcomes of OMGU in prepubertal boys with hypospadias, particularly focusing on changes observed after puberty. Only three studies of moderate quality examining long-term outcomes of oral mucosa graft urethroplasty (OMGU) in prepubertal boys with hypospadias were identified. The scarcity of high-quality evidence underscores a significant gap in our understanding of OMGU's long-term efficacy and safety in this population [11, 12]. The outcomes of OMGU were mixed, with some patients experiencing favorable results and minimal complications, while others faced significant issues, including meatal stenosis, urethral strictures, and fistulas that necessitated additional procedures. These complications suggest potential challenges in maintaining the integrity and function of the graft over time [11, 12].

Comparing OMGU outcomes to other long-term studies is challenging, as OMGU is often used in difficult cases and re-operations. Studies on preputial grafts and urethral plate reconstruction have shown generally good functional and cosmetic results in long-term follow-ups. For instance, Spinoit et al. followed 474 patients who underwent prepuce-only hypospadias repair for a median of 23 years, finding that 70% of patients were satisfied with penile appearance and 75% with sexual function [13]. Similarly, Örtqvist et al. and Rynja et al. reported generally positive long-term results in their studies [14, 15]. However, the lack of comparable long-term data for OMGU and differences in case mix make direct comparisons difficult.

When referring to “long-term outcomes” in hypospadias repair, we specifically mean follow-up assessments that extend beyond puberty and into adulthood. This distinction is crucial due to the significant changes that occur in the external genitalia during puberty. Comprehensive long-term follow-up data are essential for evaluating the durability and stability of surgical outcomes over time, particularly in assessing functional and cosmetic outcomes in the post-pubertal period [13, 14]. An international registry of all hypospadias patients, like proposed in [16] would give a clear follow-up of old techniques and compare them with new ones.

During puberty, androgens, particularly testosterone and its metabolite dihydrotestosterone (DHT), significantly influence the development of male external genitalia. These hormones bind to androgen receptors, activating gene transcription and protein synthesis, which leads to tissue growth and development [17, 18]. Recent research suggests that human oral keratinocytes are sensitive to testosterone [19], indicating that oral mucosa used for urethral reconstruction may respond to hormonal changes during puberty. However, the impact of these hormonal changes on OMGU outcomes still warrants further investigation.

While our review focused primarily on complications and functional outcomes, it’s crucial to consider the patient's perspective in evaluating surgical success. The study by Nelson et al. utilized patient-reported outcomes (PROs), which are essential for assessing satisfaction and quality of life post-surgery [8]. Future studies in this field should prioritize incorporating such outcomes to provide a more comprehensive assessment of surgical success beyond just clinical measures.

Adult studies have shown generally favorable outcomes for OMGU, with success rates ranging from 80 to 90% in various series [1, 20]. These studies demonstrate the durability and functionality of oral mucosa in urethral reconstruction. However, projecting these findings to children requires caution, as the pediatric population differs in several key aspects, including growth potential, hormonal changes, and tissue characteristics.

This systematic review offers several strengths. It provides an analysis of the current studies on long-term outcomes of OMGU in prepubertal boys with hypospadias, highlighting critical gaps in knowledge and providing a clear direction for future research. The review synthesizes data from three studies. This review contributes to a better understanding of the current state of knowledge and the areas that require further investigation in the field of pediatric OMGU.

The limitations of this review include the small number of studies that could answer our research question, which affects the generalizability and reliability of the data. The studies included were found to have a serious risk of bias in various domains. The lack of standardized outcome measures across studies hinders the ability to compare different surgical approaches effectively. The retrospective and cross-sectional nature of the included studies introduces inherent biases, including selection bias, information bias, and publication bias.

## Conclusions

This systematic review identifies a significant knowledge gap regarding the long-term outcomes of OMGU in prepubertal boys with hypospadias, with current evidence insufficient to establish its efficacy and safety, particularly concerning pubertal development and post-pubertal outcomes. Future research should prioritize longitudinal studies with larger sample sizes and standardized outcome measures to address these critical gaps. Establishing a unified European database could enhance the generalizability of findings, while basic research on oral mucosa tissue properties may provide insights into its performance during puberty.

## Supplementary Information

Below is the link to the electronic supplementary material.

Supplementary file1 (DOCX 22 KB)

Supplementary file2 (DOCX 22 KB)

## Data Availability

No datasets were generated or analysed during the current study.
