# Supplementary material for: Post pubertal outcome after use of oral mucosa in urethral reconstruction for hypospadias in prepubertal boys: a systematic review
Source: Pediatr Surg Int. 2025 Feb 13;41(1):79. doi: 10.1007/s00383-025-05982-3 (PMC11825549; doi:10.1007/s00383-025-05982-3)
Supplement: Supplementary file 1 — Supplementary file1 (DOCX 22 KB) [file 383_2025_5982_MOESM1_ESM.docx]

| Section/Topic | Item | Checklist item | Reported |
| --- | --- | --- | --- |
| Title | 1 | Identify the report as a systematic review in the title | Yes |
| Abstract | 2 | Structured summary provided | Yes |
| Introduction | 3 | Rationale described | Yes |
|  | 4 | Objectives stated | Yes |
| Methods | 5 | Protocol and registration | Not mentioned |
|  | 6 | Eligibility criteria | Partially |
|  | 7 | Information sources | Yes |
|  | 8 | Search strategy | Yes |
|  | 9 | Study selection process | Yes |
|  | 10 | Data collection process | Yes |
|  | 11 | Data items | Yes |
|  | 12 | Risk of bias in individual studies | Yes |
|  | 13 | Summary measures | Not applicable |
|  | 14 | Synthesis of results | Not applicable |
|  | 15 | Risk of bias across studies | Partially |
|  | 16 | Additional analyses | Not applicable |
| Results | 17 | Study selection | Yes |
|  | 18 | Study characteristics | Yes |
|  | 19 | Risk of bias within studies | Yes |
|  | 20 | Results of individual studies | Yes |
|  | 21 | Synthesis of results | Yes |
|  | 22 | Risk of bias across studies | yes |
|  | 23 | Additional analysis | Not applicable |
| Discussion | 24 | Summary of evidence | Yes |
|  | 25 | Limitations | Yes |
|  | 26 | Conclusions | Yes |
| Funding | 27 | Funding sources/role of funders | Not mentioned |
