# Supplementary material for: Post pubertal outcome after use of oral mucosa in urethral reconstruction for hypospadias in prepubertal boys: a systematic review
Source: Pediatr Surg Int. 2025 Feb 13;41(1):79. doi: 10.1007/s00383-025-05982-3 (PMC11825549; doi:10.1007/s00383-025-05982-3)
Supplement: Supplementary file 2 — Supplementary file2 (DOCX 22 KB) [file 383_2025_5982_MOESM2_ESM.docx]

Supplemental data S1: search strings for Pubmed and Embase

("mouth mucosa"[MeSH Terms] OR ("mouth"[All Fields] AND "mucosa"[All Fields]) OR "mouth mucosa"[All Fields] OR ("oral"[All Fields] AND "mucosa"[All Fields]) OR "oral mucosa"[All Fields]) AND ("hypospadias"[MeSH Terms] OR "hypospadias"[All Fields])

("mouth mucosa"[MeSH Terms] OR ("mouth"[All Fields] AND "mucosa"[All Fields]) OR "mouth mucosa"[All Fields] OR ("oral"[All Fields] AND "mucosa"[All Fields]) OR "oral mucosa"[All Fields]) AND ("urethra"[MeSH Terms] OR "urethra"[All Fields] OR "urethral"[All Fields]) AND ("reconstructive surgical procedures"[MeSH Terms] OR ("reconstructive"[All Fields] AND "surgical"[All Fields] AND "procedures"[All Fields]) OR "reconstructive surgical procedures"[All Fields] OR "reconstruction"[All Fields]) AND ("pediatrics"[MeSH Terms] OR "pediatrics"[All Fields] OR "pediatric"[All Fields])

("mouth mucosa"[MeSH Terms] OR ("mouth"[All Fields] AND "mucosa"[All Fields]) OR "mouth mucosa"[All Fields] OR ("oral"[All Fields] AND "mucosa"[All Fields]) OR "oral mucosa"[All Fields]) AND ("urethra"[MeSH Terms] OR "urethra"[All Fields] OR "urethral"[All Fields]) AND ("reconstructive surgical procedures"[MeSH Terms] OR ("reconstructive"[All Fields] AND "surgical"[All Fields] AND "procedures"[All Fields]) OR "reconstructive surgical procedures"[All Fields] OR "reconstruction"[All Fields]) AND ("child"[MeSH Terms] OR "child"[All Fields] OR "children"[All Fields])
